# Predicting Optical Coherence Tomography-Derived High Myopia Grades From Fundus Photographs Using Deep Learning

**DOI:** 10.3389/fmed.2022.842680

**Published:** 2022-03-03

**Authors:** Zhenquan Wu, Wenjia Cai, Hai Xie, Shida Chen, Yanbing Wang, Baiying Lei, Yingfeng Zheng, Lin Lu

**Affiliations:** ^1^State Key Laboratory of Ophthalmology, Zhongshan Ophthalmic Center, Sun Yat-sen University, Guangzhou, China; ^2^Health Science Center, School of Biomedical Engineering, Shenzhen University, Shenzhen, China

**Keywords:** artificial intelligence, high myopia, fundus photographs, optical coherence tomography, deep learning

## Abstract

**Purpose:**

To develop an artificial intelligence (AI) system that can predict optical coherence tomography (OCT)-derived high myopia grades based on fundus photographs.

**Methods:**

In this retrospective study, 1,853 qualified fundus photographs obtained from the Zhongshan Ophthalmic Center (ZOC) were selected to develop an AI system. Three retinal specialists assessed corresponding OCT images to label the fundus photographs. We developed a novel deep learning model to detect and predict myopic maculopathy according to the atrophy (A), traction (T), and neovascularisation (N) classification and grading system. Furthermore, we compared the performance of our model with that of ophthalmologists.

**Results:**

When evaluated on the test set, the deep learning model showed an area under the receiver operating characteristic curve (AUC) of 0.969 for category A, 0.895 for category T, and 0.936 for category N. The average accuracy of each category was 92.38% (A), 85.34% (T), and 94.21% (N). Moreover, the performance of our AI system was superior to that of attending ophthalmologists and comparable to that of retinal specialists.

**Conclusion:**

Our AI system achieved performance comparable to that of retinal specialists in predicting vision-threatening conditions in high myopia via simple fundus photographs instead of fundus and OCT images. The application of this system can save the cost of patients' follow-up, and is more suitable for applications in less developed areas that only have fundus photography.

## Introduction

Myopia has been recognized as an important public health problem worldwide (1). The global number of myopic subjects will increase to 5 billion by 2050, and about 20% of them will suffer from high myopia (2). The high prevalence of myopia and high myopia leads to an increase in pathological myopia (PM), especially in East Asian countries (3). Patients with PM usually suffer from myopic maculopathy, which is one of the most common causes of irreversible blinding vision loss (4). Visually impaired people tend to have lower capacity for work and higher rate of depression, imposing a significant burden on individuals and society (5). Formulating an applicable strategy for risk stratification is conducive to surveillance and early treatment, (6, 7) but the diagnosis and assessment of myopic maculopathy in the clinic are relatively complex (8).

The atrophy, traction, and neovascularisation (ATN) classification and grading system proposed by Jorge Ruiz-Medrano is currently a common clinical diagnostic criterion, allowing a simple and systematic grading of myopic maculopathy that is both understandable and widely applicable (3). It contains three categories: atrophy (A), traction (T), and neovascularisation (N). Clinically, the diagnosis of category A can depend on fundus photographs, but the diagnosis of categories T and N is difficult based on fundus photographs only, and optical coherence tomography (OCT) images are also required. However, it is difficult to widely adopt OCT examinations as a routine screening method because of the high cost and the lack of equipment in primary hospitals or communities. Therefore, rapid screening and timely referral for PM is handicapped because the T and N conditions cannot be reliably detected by humans using fundus photographs alone. Ophthalmologists with sufficient experience in diagnosing and treating high myopia are also scarce in primary hospitals.

A potential solution is development of artificial intelligence (AI) technology, which has been applied to identify various ophthalmic diseases (9–16). Previous studies reported that AI systems can predict diseases from fundus photographs, such as cardiovascular risk factors and refractive error (17, 18). A recent study successfully predicted OCT-derived center-involved diabetic macular oedema (ci-DME) from fundus photographs using deep learning (19). It suggested that the AI system can discover the underlying association between the disease and the details of fundus photographs. As for high myopia, previous studies only focused on automated lesion identification based on fundus or OCT images (20, 21). Further exploration is required to allow making predictions in high myopia via two-dimensional images (fundus photographs) without three-dimensional images (OCT images).

This study aimed to develop an AI system that can predict OCT-derived ATN classification using monoscopic fundus photographs.

## Materials and Methods

This study was approved by the Ethics Committee of Zhongshan Ophthalmic Center (ZOC, Guangzhou, China) (ID: 2021KYPJ175) and the requirement of informed consent was waived in this retrospective study. And this study was performed in accordance with the principles of the Declaration of Helsinki; all private information that could identify individuals was excluded.

### Patients and Images

Patients with high myopia presenting to the retinal clinic at ZOC between January 2014 and February 2021 were reviewed and analyzed. High myopia was defined as refractive error (RE) ≤ −6.0 D or axial length (AL) ≥ 26.0 mm. None of the patients had previously undergone surgery. Patients with coexisting or previous ocular disorders, such as diabetic retinopathy, retinal vascular abnormalities, uveitis, and age-related macular degeneration, were excluded.

Fundus photographs were collected using a Topcon fundus camera (TRC-50; Topcon, Tokyo, Japan), and one single macula-centered 50°color fundus photograph was obtained for each eye. All fundus photographs were downloaded using the standard tiff or jpeg compression formats. The corresponding spectral domain OCT images (Heidelberg Engineering, Heidelberg, Germany) were collected. The OCT images with horizontal and vertical slices through the fovea were downloaded in a standard image format according to the manufacturer's software instructions, and the scan length was 6 mm. These slices can detect most vision-threatening conditions associated to high myopia. For some patients, the same eye was examined multiple times to monitor disease progression. If the interval between the two examinations exceeded 1 month and the multiple examinations showed that the disease is progressing, the images of the multiple examinations will be included as separate data in this study. There were no exclusion criteria based on age, sex, or race.

### Image Labeling Process Based on the ATN System

**Figure 1:**
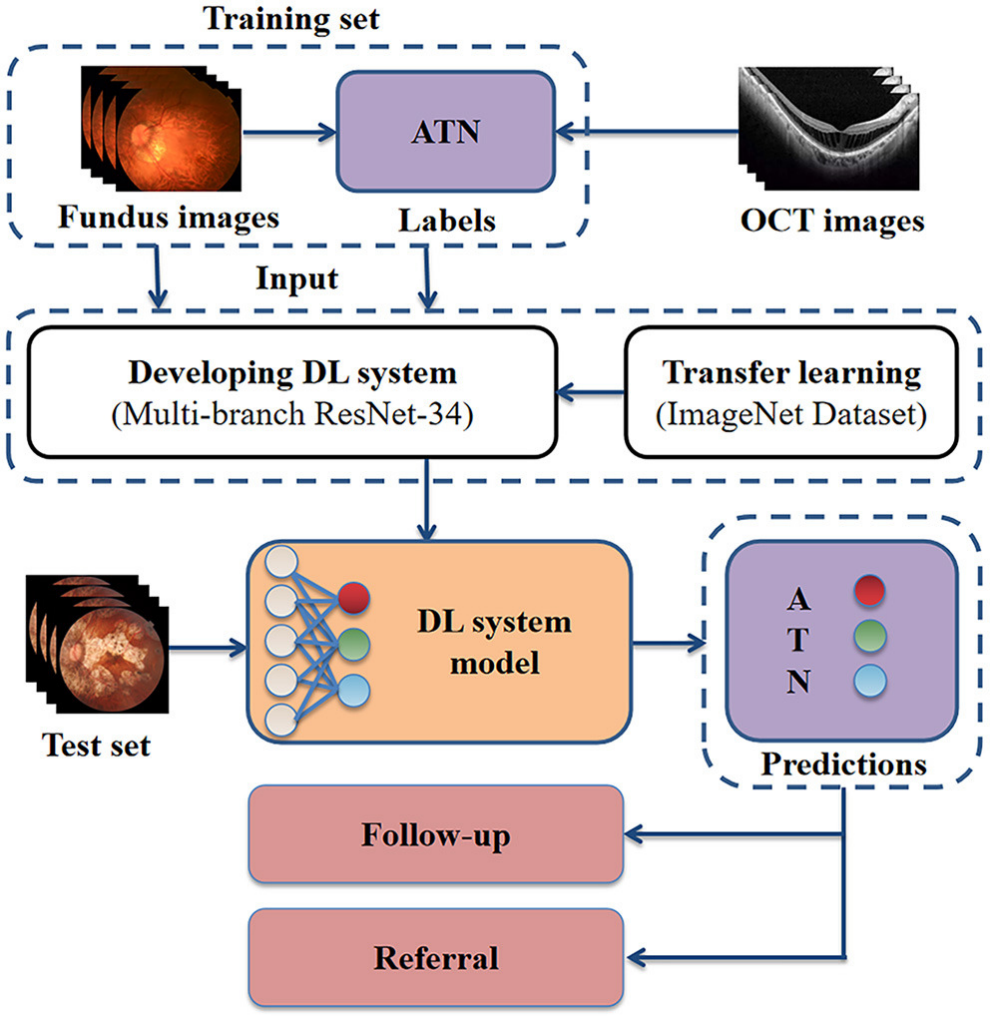
Schematic representation of our approach for developing a high myopia predictive model. Retinal specialists labeled each fundus photograph according to the corresponding OCT images. The fundus photographs with ground true labels were used for model training. For clinical application, the model receives as input fundus photographs, then outputs the predicted ATN classification. OCT, optical coherence tomography; DL, deep learning; A, atrophy; T, traction; N, neovascularisation.

**Figure 2:**
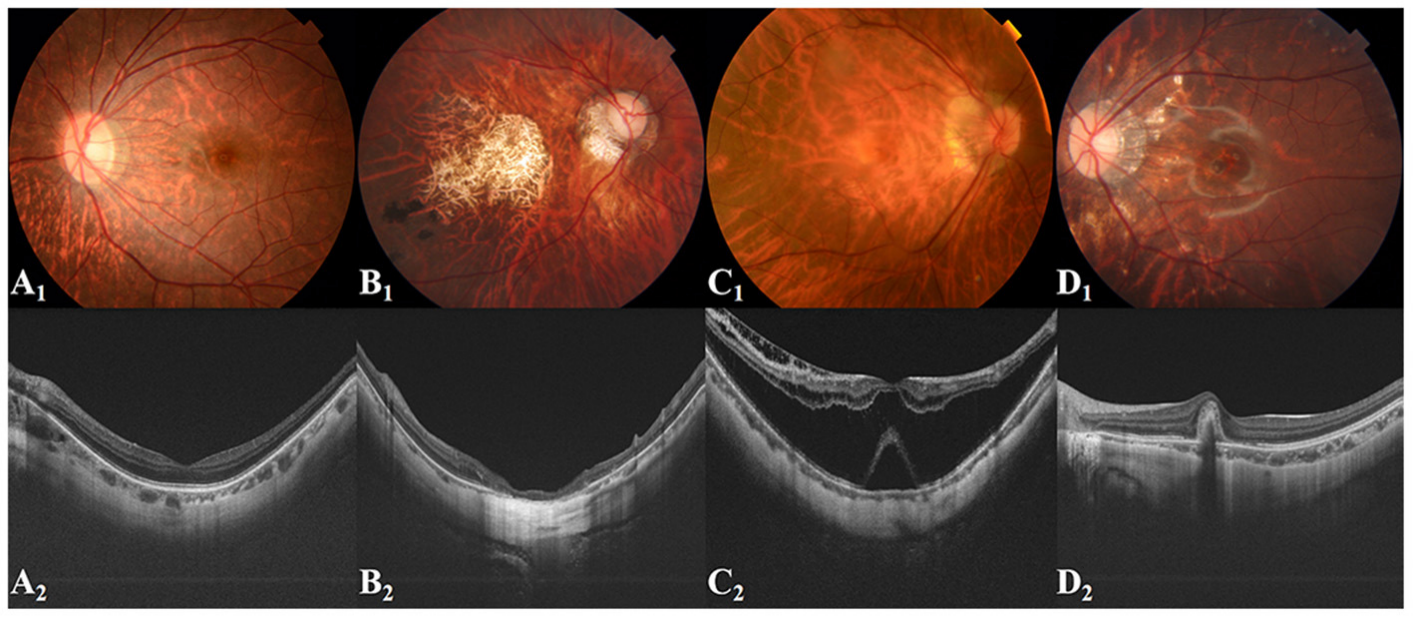
Representative fundus images and related OCT images. **(A**_**1**_**,A**_**2**_**)** Tessellated fundus; **(B**_**1**_**,B**_**2**_**)** Macular atrophy; **(C**_**1**_**,C**_**2**_**)** Retinal detachment; **(D**_**1**_**,D**_**2**_**)** PMCNV. OCT, optical coherence tomography; PMCNV, pathological myopic choroidal neovascularisation.

Three Chinese board-certified retinal specialists with over 10 years of experience were invited to label each fundus photograph according to the corresponding OCT images of each eye (Figure 1). Initial quality control was conducted for all fundus photographs. Images in which the optic disc and macula could not be identified clearly were considered poor quality and removed from the database. Duplicated images and those with incorrect magnification were also excluded. Qualified fundus photographs were used to train the AI system, whereas OCT images were used only to aid labeling. The labeling standard was consistent with the ATN system of Ruiz-Medrano et al. (3). All fundus photographs were graded separately for the A, T, and N categories and placed into the dataset accordingly. Each image was examined, discussed, and labeled until all three retinal specialists agreed on the final diagnosis. When dissent could not be resolved, another retinal specialist with over 20 years of experience was invited to the group discussion to make the final decision. Representative fundus photographs and relative OCT images are shown in Figure 2.

### Development of a Deep Learning Model

We used binary classification to simplify the ATN system, which can screen out patients who need close follow-up or treatment. Specifically, retinal atrophy (A1), macular hole or retinal detachment (T1), and pathological myopic choroidal neovascularisation (PMCNV) (N1) were detected or predicted (Table 1). In addition, we attempted to use multi-classification to divide category A into three grades, including normal fundus (no myopic retinal lesions), tessellated fundus, and retinal atrophy. The purpose of this task was to further distinguish the presence of high myopia in fundus photographs without retinal atrophy.

**Table 1:** Final classification standard.

**Atrophic component (A)**	**Tractional component (T)**	**Neovascular component (N)**
A0: No myopic retinal lesions, or tessellated fundus only	T0: No macular traction, or foveoschisis only	N0: No PMCNV
A1: Retinal atrophy	T1: MH or retinal detachment	N1: PMCNV

*PMCNV, Pathological myopic choroidal neovascularisation; MH, Macular hole*.

**Figure 3:**
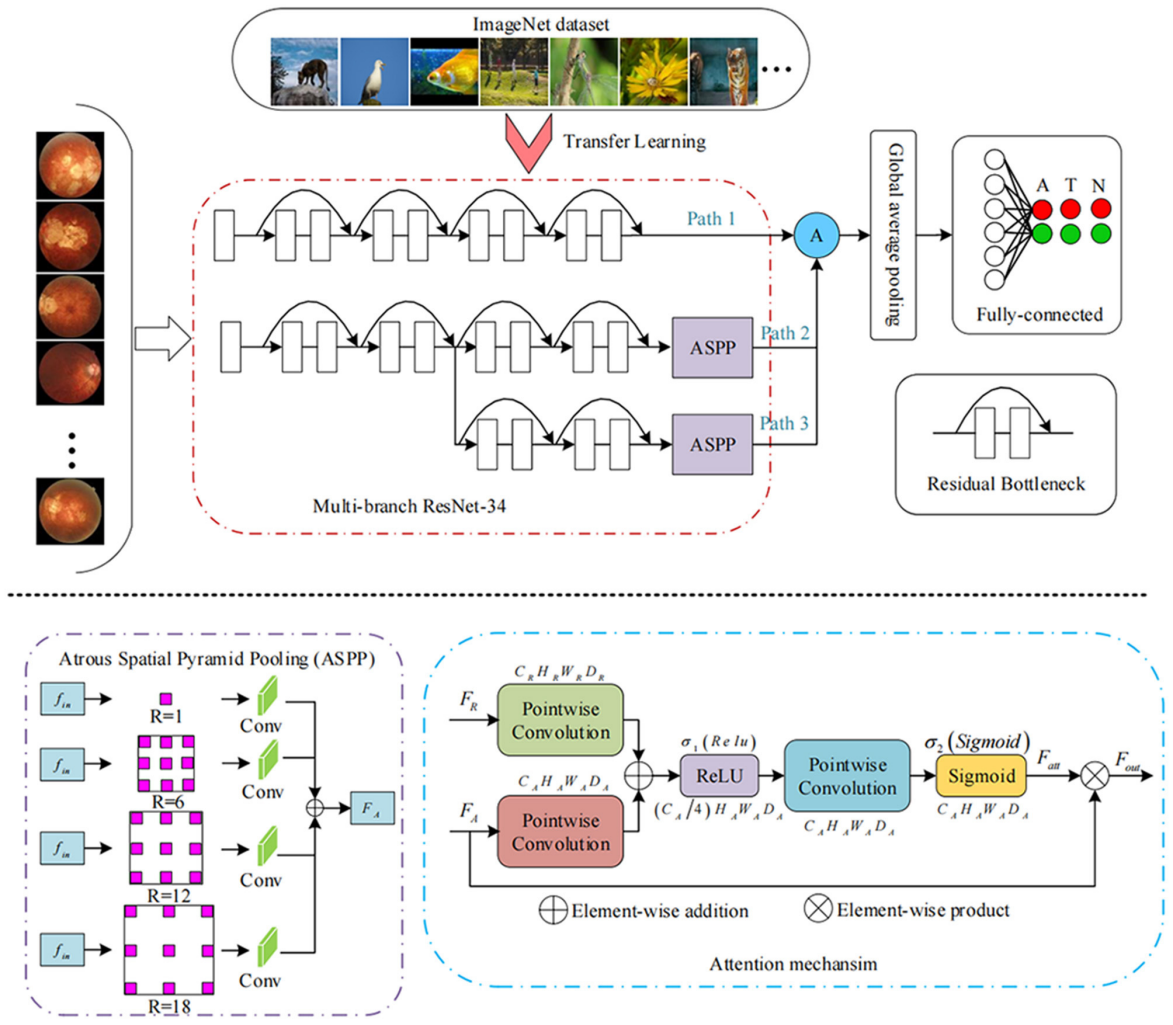
Framework of the deep learning methods.

**Figure 4:**
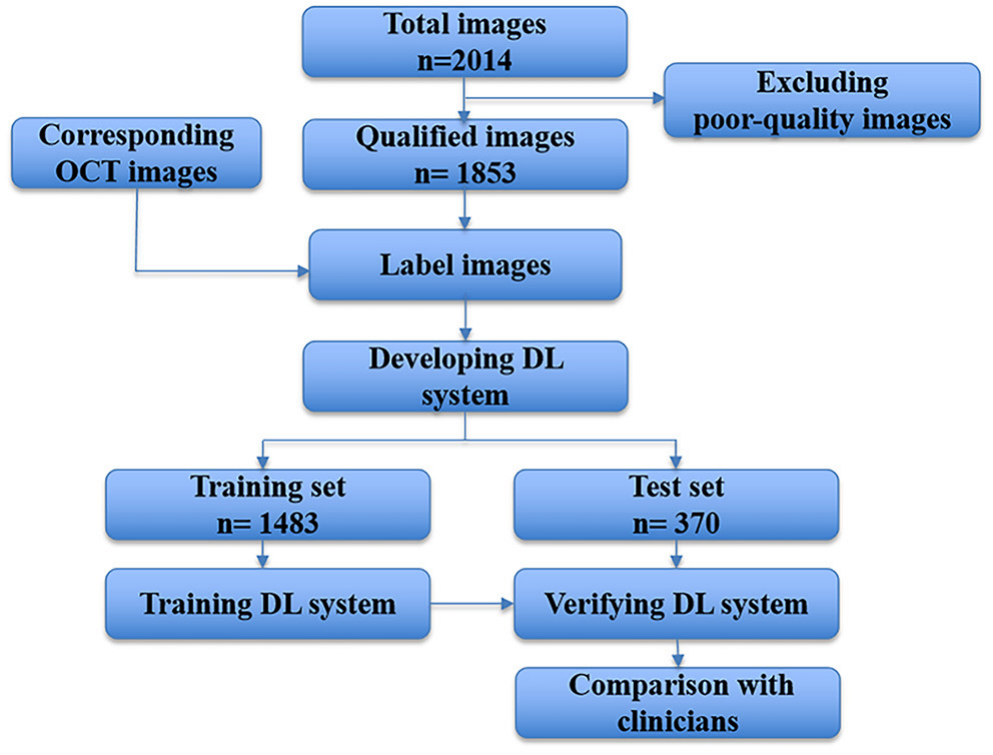
Flow chart showing the AI system development and evaluation based on fundus photographs. DL, deep learning; OCT, optical coherence tomography.

The devised framework used the residual network ResNet-34 model as the backbone to build a multi-branch ResNet-34 structure, which is utilized to extract rich high-level feature information (22, 23). We used the pre-trained parameters on the ImageNet dataset to train the designed multi-branch ResNet-34 model in transfer learning mode. The atrous spatial pyramid pooling (ASPP) module was employed to extract contextual feature information by expanding the receptive field, with the atrous rate set to 1, 6, 12, and 18. To force the network to focus on the feature extraction of the key areas, the attention module was used to refine the extracted feature by the multi-branch network so that discriminative features could be obtained. Subsequently, we used the global average pooling operation to process the extracted features. Following the pooling layer, a fully connected layer was used to map the extracted features to the category space. Finally, the entire network was trained and tested to output the predicted results using the softmax layer (22). The entire framework of the proposed deep learning model is shown in Figure 3. We used the Pytorch library as the framework to build the network. One NVIDIA TITAN XP GPU was employed to accelerate training and testing. The batch size was set to 16, and the maximum epoch of training was 60. The input images were resized to 512 × 512 pixels. The initial learning rate was set to 0.0001, which was multiplied by 0.1 every 20 epochs. Afterward, the data were randomly classified into training set and testing set in the ratio of 0.8:0.2 in each category. The flow chart in Figure 4 shows the development and evaluation of the AI system based on fundus photographs.

### Quantification and Statistical Analysis

We used accuracy, precision, sensitivity, specificity, and F1 score to evaluate the performance and stability of the classification model. Accuracy was measured by dividing the number of correct predictions by the total number of samples. Precision was measured by dividing the number of true positives by the total number of true positives and false positives. Sensitivity was determined by dividing the number of true positives by the total number of true positives and false negatives. Specificity, was determined by dividing the number of true negatives by the total number of true negatives and false positives. The F1 score was calculated as twice the product of precision and sensitivity divided by their sum and measures the balance of the positive and negative samples at the same time. For further evaluation, two retinal specialists and two attending ophthalmologists were invited to classify the images to compare with the AI system. The area under the receiver operating characteristic (ROC) curve (AUC) was calculated to assess the total performance of AI models and clinicians.

## Results

### Imaging Datasets and Clinical Characteristics of Patients

A total of 2,014 fundus photographs were collected, including 1,814 high myopia fundus photographs (704 subjects) and 200 normal fundus photographs (116 subjects). After quality control, 161 poor quality images were excluded. Finally, 1,853 fundus photographs were selected to develop and evaluate our AI system. We split the data assigning 1,483 images to the training dataset and 370 to the test dataset. The demographic characteristics of included subjects are shown in Table 2.

**Table 2:** Demographic characteristics of subjects.

**Parameters**	**High myopia**	**Normal**
Number of gradable images	1,653	200
Number of subjects	704	116
Gender (male/female)	310/394	51/65
Age (years)	52.62 ± 14.03	56.37 ± 9.23
BCVA (LogMAR)	0.57 ± 0.53	0.11 ± 0.10
AL (mm)	29.54 ± 2.44	23.09 ± 0.91
SE (D)	−11.90 ± 7.20	0.03 ± 0.99

*Data are presented as mean ± standard deviation*.

*BCVA, best corrected visual acuity; AL, Axial length; SE, spherical equivalent*.

### Performance of the AI System

**Figure 5:**
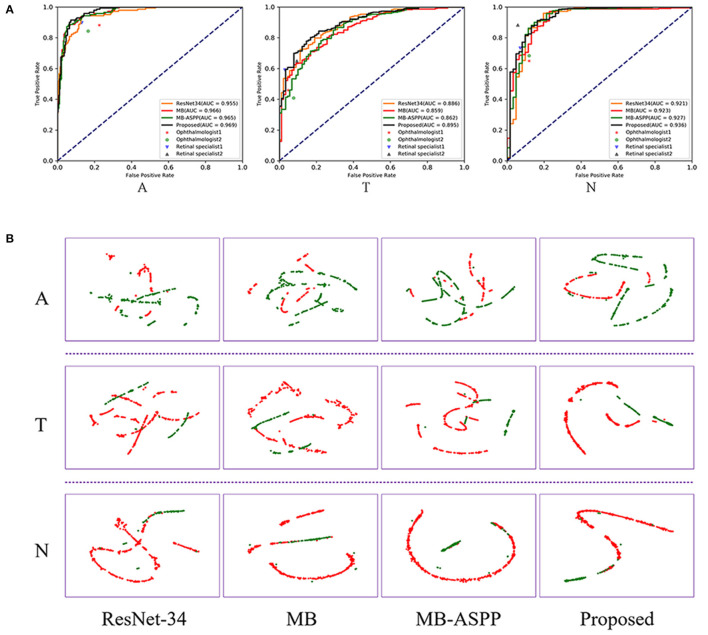
Performance of AI system in A, T, and N categories. **(A)** The comparison of different methods and clinicians using ROC curves. AUC, area under the curve; ROC, receiver operating characteristic. **(B)** The t-SNE visualization of different methods. Red and green points represent negative and positive results, respectively.

The AUCs of the proposed method were 0.969, 0.895, and 0.936 for categories A, T, and N, respectively, and the accuracies were 92.38, 85.34, and 94.21%, respectively (Figure 5A; Table 3). Compared with other methods, the proposed method achieved the highest AUC values and accuracies for all categories. Further indicators including precision, sensitivity, specificity, and F1-score are shown in Table 3. We also produced t-distributed stochastic neighbor embedding (t-SNE) visualizations for the different methods using the last extracted features (Figure 5B). Red and green points represent negative and positive results, respectively. Points of the same color were clustered, while those of different color were separated, indicating the good classification ability of the model. We can observe that the proposed method could separate the categories more easily than other methods.

**Table 3:** Comparison of different methods in the testing dataset (%).

	**Method**	**Accuracy**	**Precision**	**Sensitivity**	**Specificity**	**F1-score**
A	ResNet-34	90.71	89.36	90.03	87.94	89.68
	MB	91.43	90.19	90.74	88.65	90.46
	MB-ASPP	92.14	91.25	91.10	87.94	91.18
	Proposed	**92.38**	**91.07**	**92.16**	**91.48**	**91.57**
T	ResNet-34	83.51	81.24	68.52	96.24	71.66
	MB	82.72	76.46	71.92	94.18	73.67
	MB-ASPP	84.55	80.67	72.72	94.88	75.40
	Proposed	**85.34**	**83.58**	**72.45**	**96.58**	**75.78**
N	ResNet-34	92.63	88.29	83.02	96.87	85.34
	MB	93.68	89.51	86.29	97.18	87.79
	MB-ASPP	93.95	90.83	85.79	97.81	88.05
	Proposed	**94.21**	**91.69**	**85.94**	**98.12**	**88.48**

*ResNet-34, original ResNet-34 model; MB, Multi-branch ResNet-34 model; MB-ASPP, Multi-branch ResNet-34 & ASPP model; Proposed, Multi-branch ResNet-34 & ASPP & Attention model. The bold values represent the results of our proposed method*.

**Figure 6:**
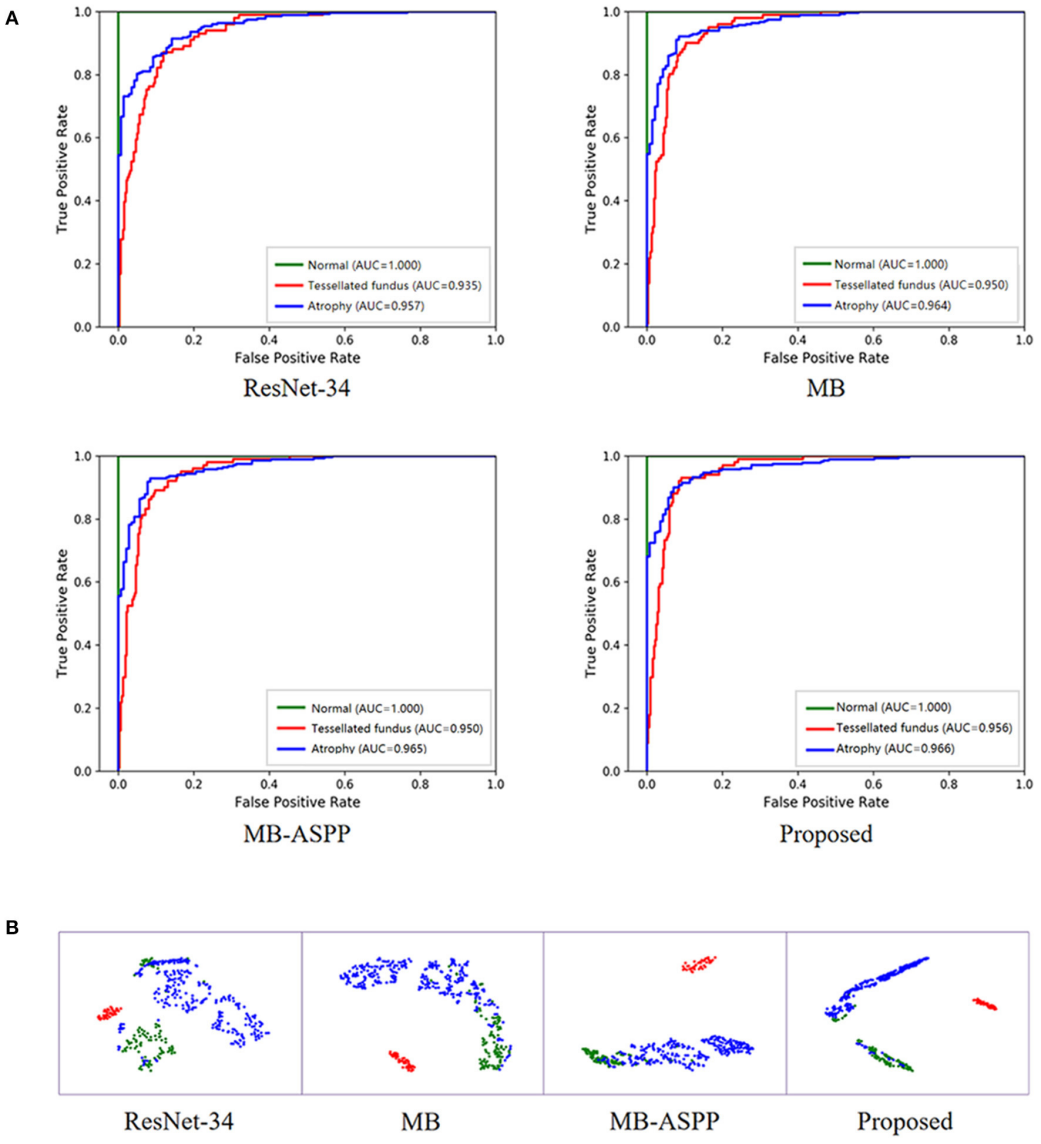
Performance of AI system in category A with three sub-grades. **(A)** The ROC curves of different methods for category A with three sub-grades. AUC, area under the curve; ROC, receiver operating characteristic. **(B)** The t-SNE visualization of different methods for category A with three sub-grades. Red, blue, and green points represent the normal fundus (no myopic retinal lesions), tessellated fundus, and retinal atrophy, respectively.

In addition, we trained the model to perform a multi-classification task for category A. Compared with the binary classification model, this multiclass model can distinguish tessellated fundus independently, which is more conducive to the identification of early lesions. The accuracies of the four methods (ResNet-34, MB, MB-ASPP, and Proposed method) were 89.05, 90.71, 90.95, and 91.67%, respectively. Figure 6A shows the AUCs of the different methods for the classification of category A with three sub-grades. The AUCs of the proposed method were 1.000, 0.956, and 0.966 in normal fundus, tessellated fundus, and retinal atrophy, respectively. The results indicate that proposed method had the best performance. Figure 6B shows the t-SNE visualizations with respect to the different methods. The proposed method could separate the categories more easily than other methods.

### Comparison of the Performance of the AI System With That of Clinicians

We compared the classification performance of the AI system with that of ophthalmologists, including two retinal specialists and two attending ophthalmologists. To illustrate the superiority of the proposed method, we plotted the ROC curves and marked the operating points of the four ophthalmologists as well (Figure 5A). The average accuracies of the two attending ophthalmologists were 84.46, 75.14, and 84.59% for categories A, T, and N, respectively; while those of the two retinal specialists were 90.00, 82.03, and 91.49%, respectively. When compared with clinicians, the proposed method performed better than attending ophthalmologists and comparably to retinal specialists.

## Discussion

Previous studies have shown that AI technology can use fundus photographs to predict cardiovascular risk, refractive errors, and ci-DME, significantly outperforming specialists (17–19). Varadarajan et al. reported that their AI system, applied fundus photographs to predict the presence of ci-DME, achieved 85% sensitivity and 80% specificity. Compared with the AI system, retinal specialists have similar sensitivities (82–85%), but only half the specificity (19). In the current study, we used fundus photography to predict the presence of treatment-requiring conditions (macular hole, retinal detachment, and PMCNV), and also achieved high accuracy in categories T (85.34%) and N (94.21%). The performance was greater than that of attending ophthalmologists and comparable to that of retinal specialists. Our system can pick up more occult lesions which might be overlooked by ophthalmologists based on fundus photographs alone. The application of this model in less developed areas is expected to solve the problem of inadequate equipment and doctors in primary hospitals or communities.

In previous researches, Li et al. (24) developed an AI model with 5505 OCT images to identify vision-threatening conditions (retinoschisis, macular hole, retinal detachment, and PMCNV) in patients with high myopia and achieved reliable sensitivities and specificities. The OCT images can clearly reveal retinal traction and neovascularisation because of the cross-sectional information, but the cost is relatively high, and there is no such equipment in many primary hospitals or communities. Therefore, it is not suitable for long-term follow-up of myopia patients in less developed areas. In this study, the AI system we developed to predict ATN classification of high myopia via fundus photographs without OCT images is comparable in performance to retinal specialists. This work demonstrates the potential of AI technology to enable diagnostics on inexpensive equipment, replacing previously expensive equipment.

Automatic diagnosis of retinal detachment and/or PMCNV by fundus photographs has been reported in some studies (25, 26). Hemelings et al. developed an algorithm for PM detection using 400 fundus photographs, and the F1 score for retinal detachment was 0.71 (25). However, it is a challenge to identify minimal changes in early stage of myopic maculopathy using only fundus photographs for both ophthalmologists and AI system. In the current study, retinal specialists labeled the fundus photographs by referring to the results of the corresponding OCT images. Some minimal lesions, which were latent on fundus photographs but present obviously on corresponding OCT images, were also included in the training dataset. Therefore, our system can pick up more details (all kinds of macular hole, retinal detachment, and PMCNV) than ophthalmologists. The connection between lesion features and results might have been built during the training process, allowing the achievement of higher predictive ability.

Some researchers have performed automatic identification and segmentation of myopic lesions based on fundus photographs, and achieved promising performance (25–28). Tan et al. developed an algorithm which achieved high diagnostic performance, with an AUC of 0.913 for high myopia and 0.969 for myopic maculopathy (26). Baid et al. developed another algorithm for PM detection and achieved an AUC of 0.99 (28). However, these studies only made a preliminary diagnosis of PM or focused on identifying one or two categories of myopic maculopathy. Our AI system can simultaneously predict atrophy, traction, and neovascularisation. Using our AI system, patients with macular hole and/or RD will be accurately predicted and referred to retinal specialists for timely surgery. PMCNV will also be predicted, and the patient referred for further examination to formulate a plan for anti-vascular endothelial growth factor treatment. Regular follow-up is advised to monitor disease progression in patients with tessellated fundus or retinal atrophy alone. Therefore, primary hospitals can achieve risk stratification and promptly detect and refer patients requiring treatment, which helps patients receive treatment in time to restore vision.

Due to the large population of patients with myopia, it is challenging for ophthalmologists to conduct large-scale screenings (5, 29). If all patients with myopia were to be referred to advanced hospitals for diagnosis or follow-up, they could overwhelm the medical system. Therefore, there is an urgent need to establish an effective community-based myopia-screening system. The non-expert-dependent AI system we developed has important clinical value, as it can reduce the large influx of patients to advanced hospitals and reduce individual and social costs. It can also be convenient for patients who require long-term follow-up. In addition, the application of this AI system in primary hospitals and healthcare institutions during the COVID-19 pandemic will reduce the concentration in large hospitals, and thus the risk of infection.

This study had some limitations. First, the ATN classification in this study has been simplified. However, the system can accurately identify patients who need referral and can save the cost of patient follow-up. Second, all images in our study were collected from the same hospital. Images from multiple centers could be obtained as external data to further improve and assess our AI system. In addition, this AI system can detect PMCNV, but it is difficult to estimate the activity of the lesion. This problem could be solved by collecting more images from the long-term follow-up of patients with PMCNV to compare the different features over time.

In conclusion, the AI system we developed can predict ATN classification of high myopia using the cheaper and more widely available fundus photographs, with performance comparable to that of retinal specialists. Its clinical applications would provide a comprehensive diagnosis to triage and referral and reduce the individual and social costs. Such promising performance recommends its extensive application as a large-scale screening tool in primary medical institutions.

## Data Availability Statement

The raw data supporting the conclusions of this article will be made available by the authors, without undue reservation.

## Ethics Statement

The studies involving human participants were reviewed and approved by the Ethics Committee of Zhongshan Ophthalmic Center (ZOC, Guangzhou, China) (ID: 2021KYPJ175). Written informed consent was not required for this study, in accordance with the local legislation and institutional requirements.

## Author Contributions

ZW, WC, and SC: conception and design. LL: funding obtainment and provision of data. ZW, SC, and YW: collection and assembly of data. HX and BL: data analysis and interpretation. YZ, LL, and SC: manuscript revision. All authors wrote the manuscript and approved the final version of the manuscript.

## Funding

This work was supported by National Natural Science Foundation of China (No.62106153) and Guangdong Province Science and Technology Projects (No.3030901010066).

## Conflict of Interest

The authors declare that the research was conducted in the absence of any commercial or financial relationships that could be construed as a potential conflict of interest.

## Publisher's Note

All claims expressed in this article are solely those of the authors and do not necessarily represent those of their affiliated organizations, or those of the publisher, the editors and the reviewers. Any product that may be evaluated in this article, or claim that may be made by its manufacturer, is not guaranteed or endorsed by the publisher.
